# Systematic Overview of Aristolochic Acids: Nephrotoxicity, Carcinogenicity, and Underlying Mechanisms

**DOI:** 10.3389/fphar.2019.00648

**Published:** 2019-06-11

**Authors:** Jiayin Han, Zhong Xian, Yushi Zhang, Jing Liu, Aihua Liang

**Affiliations:** Key Laboratory of Beijing for Identification and Safety Evaluation of Chinese Medicine, Institute of Chinese Materia Medica, China Academy of Chinese Medical Sciences, Beijing, China

**Keywords:** aristolochic acids, aristolochic acid nephropathy, Balkan endemic nephropathy, upper tract urothelial carcinoma, mechanisms of nephrotoxicity, carcinogenicity of aristolochic acids

## Abstract

Aristolochic acids (AAs) are a group of toxins commonly present in the plants of genus *Aristolochia* and *Asarum*, which are spread all over the world. Since the 1990s, AA-induced nephropathy (AAN) and upper tract urothelial carcinoma (UTUC) have been reported in many countries. The underlying mechanisms of AAN and AA-induced UTUC have been extensively investigated. AA-derived DNA adducts are recognized as specific biomarkers of AA exposure, and a mutational signature predominantly characterized by A→T transversions has been detected in AA-induced UTUC tumor tissues. In addition, various enzymes and organic anion transporters are involved in AA-induced adverse reactions. The progressive lesions and mutational events initiated by AAs are irreversible, and no effective therapeutic regimen for AAN and AA-induced UTUC has been established until now. Because of several warnings on the toxic effects of AAs by the US Food and Drug Administration and the regulatory authorities of some other countries, the sale and use of AA-containing products have been banned or restricted in most countries. However, AA-related adverse events still occur, especially in the Asian and Balkan regions. Therefore, the use of AA-containing herbal remedies and the consumption of food contaminated by AAs still carry high risk. More strict precautions should be taken to protect the public from AA exposure.

## Introduction

Aristolochic acids (AAs) are identified as a group of toxins that can cause end-stage renal failure associated with urothelial carcinoma. In 1992, a high prevalence of kidney disease accompanied by urothelial carcinoma in female patients ingesting slimming pills raised worldwide attention to the high nephrotoxic and carcinogenic potential of AAs. Subsequently, Balkan endemic nephropathy (BEN) has also been found to be associated with the exposure to AAs. Since then, different studies have addressed the characterization and quantitation of AA analogs in plants and products, and the underlying mechanisms involved in the adverse reactions of AAs have been broadly described (Zhou et al., 2019).

## Aristolochic Acids

**Figure 1:**
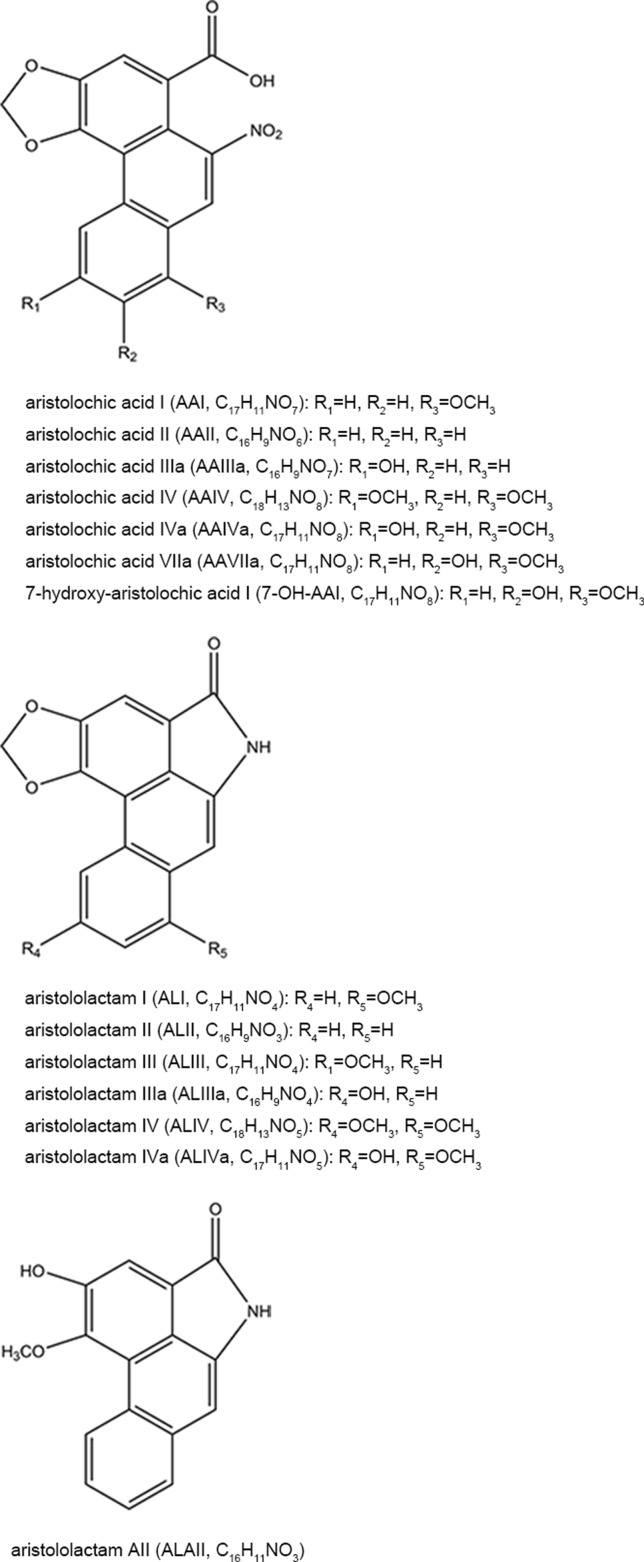
Chemical structures of some typical AA analogs.

AAs are abundant in the plants of genus *Aristolochia* and *Asarum*, which are spread all over the world (Hashimoto et al., 1999; Wooltorton, 2004; Liang et al., 2017). So far, more than 178 AA analogs have been isolated from natural sources (Michl et al., 2014), in which at least seven species of *Aristolochia*, including *Aristolochia indica* L. (Asia), *A. serpentaria* L. (North America), *A. debilis* Sieb and Zucch. (China), *A. acuminata* Lam (India), *A. trilobata* L. (Central/South America, Caribbean), *A. clematitis* L. (Europe), and *A. bracteolata* Lam. (Africa) (Heinrich et al., 2009), as well as four species of *Asarum*, including *Asarum heteropoides* f. *mandshuricum* (Maxim). Kitag and *A. sieboldii* Miq (China), *A. europaeum* L. (Europe), and *A. canadense* L (Canada and USA) (Michl et al., 2017) are used medicinally. Herbs or products containing AAs are commonly used for treating cold, headache, aphthous stomatitis, inflammatory diseases, snake bites, and sexual problems (Li et al., 2010; Kuo et al., 2012; Michl et al., 2013; Bhattacharjee et al., 2017; Liang et al., 2017). Since nephrotoxicity and carcinogenicity of AAs have been recognized, the US Food and Drug Administration and regulatory authorities of some other countries have issued alerts against the use and import of products containing parts of *Aristolochia.* The sale and use of AA-containing products are banned or restricted in most countries. However, in the US and Europe, herbal supplements containing AAs could be easily purchased through the Internet (Gold and Slone, 2003; Schaneberg and Khan, 2004; Michl et al., 2013). In addition, in China and some Asian countries, herbal remedies and products containing herb preparations from *Aristolochia* and *Asarum* are still used, and millions of people may be at risk of developing AA-related disease (Hu et al., 2004; Grollman, 2013; Rosenquist and Grollman, 2016). Studies have been performed to assess the AA content in different plants and products. Some typical AA analogs (**Figure 1**) obtained from plants and Chinese patent medicines are listed in **Tables 1** and **2**.

**Table 1:** Contents of AA analogs in plants.

Botanical name	Plant part	AAI	AAII	AA IIIa	AAIV	AA IVa	AA VIIa	7-OH-AAI	ALI	ALII	ALIII	ALIIIa	ALIV	ALIVa	AL AII
*Aristolochia albida* (Michl et al., 2016)	Root	1.346	1.413	0.402	NR	0.055	NR	NR	0.007	NR	NR	NR	NR	NR	NR
*Aristolochia argentina* (Michl et al., 2016)	Stem	0.085	0.156	−	NR	0.003	NR	NR	−	NR	NR	NR	NR	NR	NR
*Aristolochia austroszechuanica* (Zhou et al., 2008)	Root or root tuber	1.050	NR	NR	NR	NR	NR	NR	NR	NR	NR	NR	NR	NR	NR
*Aristolochia baetica* (Michl et al., 2016)	Leaf	0.086	0.073	0.002	NR	0.001	NR	NR	−	NR	NR	NR	NR	NR	NR
*Aristolochia californica* (Michl et al., 2016)	Stem	0.802	0.070	0.002	NR	0.008	NR	NR	0.013	NR	NR	NR	NR	NR	NR
*Aristolochia chamissonis* (Michl et al., 2016)	Leaf	0.682	−	−	NR	0.004	NR	NR	0.003	NR	NR	NR	NR	NR	NR
*Aristolochia cinnabarina* (Zhou et al., 2008; Zhang et al., 2013b; Wang and Chan, 2014; Yu et al., 2016; Li et al., 2017)	Root	0.887–12.098	0.659–5.076	0.841	NR	0.246	NR	NR	−	+	NR	+	NR	NR	−
*Aristolochia clematitis* (Michl et al., 2016)	Root	1.496	2.557	0.048	NR	0.014	NR	NR	0.002	NR	NR	NR	NR	NR	NR
*Aristolochia contorta* (Zhai et al., 2006; Yuan et al., 2007a; Yuan et al., 2007b; Yuan et al., 2008; Xu et al., 2010; Liu et al., 2011; Xu et al., 2013; Li et al., 2017; Mao et al., 2017; Ding et al., 2018)	Fruit	0.034–4.695	0.010–0.574	0.006–2.081	NR	0.019–1.370	0.019–0.610	0.765–0.902	0.071–0.446	0.012–0.061	<LOQ	0.021	<LOQ	0.045–1.080	0.010–0.048
*Aristolochia contorta* (Mao et al., 2017)	Seed	0.840–2.293	0.014–0.132	NR	NR	NR	NR	NR	NR	NR	NR	NR	NR	NR	NR
*Aristolochia contorta* (Mohamed et al., 1999; Wei et al., 2005; Zhai et al., 2006; Zhang et al., 2006; Kuo et al., 2010)	Root	0.511–6.421	0.029–6.108	0.462	NR	0.375–0.688	NR	NR	0.017–0.020	0.015–0.021	NR	NR	NR	NR	NR
*Aristolochia contorta* (Zhai et al., 2006; Yuan et al., 2007a; Yuan et al., 2008)	Herb	0.127–10.460	0.034–6.325	0.375–1.085	NR	0.258–0.308	NR	1.030–1.150	0.021	−	NR	0.026–0.105	NR	0.010	0.015
*Aristolochia cucurbitifolia* (Michl et al., 2016)	Leaf	1.107	0.122	0.004	NR	0.009	NR	NR	0.122	NR	NR	NR	NR	NR	NR
*Aristolochia cymbifera* (Michl et al., 2016)	Stem	0.016	0.127	0.005	NR	0.004	NR	NR	−	NR	NR	NR	NR	NR	NR
*Aristolochia debilis* (Hashimoto et al., 1998; Liu et al., 2005; Xu et al., 2013; Li et al., 2017)	Fruit	0.299–1.532	0.064–0.524	0.369–1.179	NR	0.030–0.240	0.318–0.872	NR	0.027–0.462	0.017–0.046	NR	NR	NR	NR	NR
*Aristolochia debilis* (Michl et al., 2016)	Stem	0.012–0.035	−	0.211	NR	0.024–0.111	NR	NR	0.004–0.006	NR	NR	NR	NR	NR	NR
*Aristolochia debilis* (Mohamed et al., 1999; Liu et al., 2005; Yuan et al., 2007a; Yuan et al., 2007b; Kuo et al., 2010; Kong et al., 2015; Li et al., 2017; Ding et al., 2018)	Root	0.078–2.610	0.013–0.875	0.004–1.400	0.120–0.180	0.002–1.750	NR	0.284–0.615	0.007–0.023	0.004–0.271	0.011–0.016	<LOD	0.096	0.096	0.102–0.285
*Aristolochia debilis* (Yuan et al., 2007b)	Herb	0.175	0.039	0.481	NR	0.245	NR	1.290	NR	<LOQ	0.016	NR	<LOQ	NR	NR
*Aritolochia elegans* (Jou et al., 2004)	NR	+	+	N	+	+	NR	NR	N	NR	NR	NR	NR	NR	+
*Aristolochia fangchi* (Kuo et al., 2010)	Fruit	0.945	0.050	NR	NR	NR	NR	NR	NR	NR	NR	NR	NR	NR	NR
*Aristolochia fimbriata* (Michl et al., 2016)	Stem	0.180	−	−	NR	0.006	NR	NR	0.016	NR	NR	NR	NR	NR	NR
*Aristolochia fontanesii* (Michl et al., 2016)	Leaf	0.855	0.102	0.106	NR	0.092	NR	NR	0.002	NR	NR	NR	NR	NR	NR
*Aritolochia foveolata* (Jou et al., 2004)	NR	+	+	+	+	+	NR	NR	+	NR	NR	NR	NR	NR	N
*Aristolochia gibertii* (Michl et al., 2016)	Leaf	0.050	1.875	0.003	NR	−	NR	NR	−	NR	NR	NR	NR	NR	NR
*Aristolochia grandiflora* (Michl et al., 2016)	Root	0.066	−	0.148	NR	0.049	NR	NR	0.028	NR	NR	NR	NR	NR	NR
*Aristolochia guentheri* (Michl et al., 2016)	Stem	0.002–0.005	−	−	NR	0.017	NR	NR	0.057	NR	NR	NR	NR	NR	NR
*Aristolochia heterophylla* (Mohamed et al., 1999; Jou et al., 2003; Zhou et al., 2008)	Stems and roots	1.640–3.260	+	+	NR	+	NR	NR	NR	NR	NR	NR	NR	NR	NR
*Aristolochia heterophylla* (Zhou et al., 2008)	Root or root tuber	1.320–4.450	NR	NR	NR	NR	NR	NR	NR	NR	NR	NR	NR	NR	NR
*Aristolochia indica* (Michl et al., 2016)	Root	0.818	0.239	0.117	NR	0.029	NR	NR	0.018	NR	NR	NR	NR	NR	NR
*Aristolochia kaempferi* (Michl et al., 2016)	Stem	1.202	1.261	0.017	NR	0.023	NR	NR	−	NR	NR	NR	NR	NR	NR
*Aristolochia labiate* (Michl et al., 2016)	Leaf	0.003	−	−	NR	−	NR	NR	−	NR	NR	NR	NR	NR	NR
*Aristolochia lagesinan* (Michl et al., 2016)	Stem	0.008	−	−	NR	−	NR	NR	0.034	NR	NR	NR	NR	NR	NR
*Aristolochia littoralis* (Michl et al., 2016)	Root	0.070	0.048	0.260	NR	0.097	NR	NR	0.034	NR	NR	NR	NR	NR	NR
*Aristolochia liukiensis* (Michl et al., 2016)	Stem	0.708	0.176	0.167	NR	0.094	NR	NR	0.001	NR	NR	NR	NR	NR	NR
*Aristolochia macrophylla* (Michl et al., 2016)	Stem	0.014	0.000	0.009	NR	0.008	NR	NR	−	NR	NR	NR	NR	NR	NR
*Aristolochia maurorum* (Michl et al., 2016)	Leaf	0.140	−	0.002	NR	0.006	NR	NR	0.003	NR	NR	NR	NR	NR	NR
*Aristolochia manshuirensis* (Hashimoto et al., 1998; Liu et al., 2005; Zhai et al., 2006; Yuan et al., 2007a; Yuan et al., 2007b; Han et al., 2008; Yuan et al., 2008; Kong et al., 2015; Yu et al., 2016; Li et al., 2017)	Stem	0.310–10.850	0.130–2.977	0.050–0.652	0.350–1.230	0.090–0.497	NR	<LOD	0.000–0.002	0.002–0.006	0.000–0.002	0.098	<LOD	<LOD	<LOD
*Aristolochia manshuirensis* (Michl et al., 2016)	Leaf	0.938–1.019	1.317–1.673	0.002–0.009	NR	0.000–0.009	NR	NR	0.001–0.004	NR	NR	NR	NR	NR	NR
*Aristolochia maxima* (Michl et al., 2016)	Root	2.151–2.467	0.540–1.438	0.022–0.024	NR	0.017–0.020	NR	NR	0.001–0.004	NR	NR	NR	NR	NR	NR
*Aristolochia mollissima* (Liu et al., 2003; Yuan et al., 2007a; Yuan et al., 2007b; Han et al., 2008; Yuan et al., 2008; Yu et al., 2016)	Herb	0.106–2.650	0.022–0.038	0.025–0.158	NR	0.041–0.058	NR	0.092–0.108	−	<LOD	<LOD	<LOQ	<LOD	0.010	<LOQ
*Aristolochia mollissima* (Zhou et al., 2008)	Aerial part	0.050	NR	NR	NR	NR	NR	NR	NR	NR	NR	NR	NR	NR	NR
*Aristolochia mollissima* (Mohamed et al., 1999)	Stem and root	0.465	NR	NR	NR	NR	NR	NR	NR	NR	NR	NR	NR	NR	NR
*Aristolochia mollissima* (Michl et al., 2016)	Leaf	1.234	−	0.001	NR	0.006	NR	NR	0.001	NR	NR	NR	NR	NR	NR
*Aristolochia moupinensis* (Michl et al., 2016)	Leaf	1.164	0.140	0.005	NR	0.038	NR	NR	0.002	NR	NR	NR	NR	NR	NR
*Aristolochia moupinensis* (Zhou et al., 2008)	Root or root tuber	0.540–2.780	NR	NR	NR	NR	NR	NR	NR	NR	NR	NR	NR	NR	NR
*Aristolochia moupinensis* (Zhou et al., 2008)	Stem	0.540–2.150	NR	NR	NR	NR	NR	NR	NR	NR	NR	NR	NR	NR	NR
*Aristolochia odoratissima* (Michl et al., 2016)	Leaf	0.054	−	−	NR	0.003	NR	NR	−	NR	NR	NR	NR	NR	NR
*Aristolochia ovalifolia* (Michl et al., 2016)	Leaf	0.419	−	−	NR	0.001	NR	NR	0.013	NR	NR	NR	NR	NR	NR
*Aristolochia paucinervis* (Michl et al., 2016)	Fruit	1.597	0.931	0.034	NR	0.055	NR	NR	0.054	NR	NR	NR	NR	NR	NR
*Aristolochia pothieri* (Michl et al., 2016)	Leaf	−	−	−	NR	−	NR	NR	0.114	NR	NR	NR	NR	NR	NR
*Aristolochia ringens* (Michl et al., 2016)	Root	0.668	0.138	−	NR	0.026	NR	NR	0.002	NR	NR	NR	NR	NR	NR
*Aristolochia rotunda* (Michl et al., 2016)	Root	1.629	1.518	0.159	NR	0.052	NR	NR	0.005	NR	NR	NR	NR	NR	NR
*Aristolochia sempervirens* (Michl et al., 2016)	Leaf	0.676	0.073	0.007	NR	0.028	NR	NR	0.005	NR	NR	NR	NR	NR	NR
*Aristolochia serpentaria* (Michl et al., 2016)	Fruit	0.992	0.077	0.002	NR	0.013	NR	NR	0.017	NR	NR	NR	NR	NR	NR
*Aritolochia shimadi* (Jou et al., 2004)	NR	+	+	+	+	+	NR	NR	N	NR	NR	NR	NR	NR	N
*Aristolochia tagala* (Michl et al., 2016)	Root	1.347	0.090	0.243	NR	0.121	NR	NR	0.006	NR	NR	NR	NR	NR	NR
*Aristolochia taliscana* (Michl et al., 2016)	Stem	0.010	−	−	NR	−	NR	NR	−	NR	NR	NR	NR	NR	NR
*Aristolochia tomentosa* (Michl et al., 2016)	Stem	1.047	0.370	0.029	NR	0.023	NR	NR	0.001	NR	NR	NR	NR	NR	NR
*Aristolochia triangularis* (Michl et al., 2016)	Stem	0.025	−	0.001	NR	0.032	NR	NR	0.005	NR	NR	NR	NR	NR	NR
*Aristolochia trilobata* (Michl et al., 2016)	Stem	0.435	0.157	0.109	NR	0.017	NR	NR	0.012	NR	NR	NR	NR	NR	NR
*Aristolochia westlandii* (Michl et al., 2016)	Stem	0.001	−	−	NR	−	NR	NR	−	NR	NR	NR	NR	NR	NR
*Aristolochia zollingeriana* (Michl et al., 2016)	Leaf	0.945–1.189	1.745–2.289	0.033–0.052	NR	0.008–0.010	NR	NR	0.004–0.011	NR	NR	NR	NR	NR	NR
*Asarum caudigelellum* (Han et al., 2008)	NR	0.150–0.220	NR	NR	NR	NR	NR	NR	NR	NR	NR	NR	NR	NR	NR
*Asarum heterotropides* (Yuan et al., 2007a; Yuan et al., 2007b; Yuan et al., 2008; Zhou et al., 2008)	Herb	0.040–0.110	0.025	0.055–0.060	0.054–0.058	0.047	NR	0.041	0.048	0.005	<LOD	0.009–0.031	<LOD	<LOD	<LOQ
*Asarum heterotropides* (Kuo et al., 2010; Wen et al., 2014)	Root and rhizome	0.008	NR	NR	NR	0.110	NR	NR	0.045	NR	NR	NR	NR	NR	NR
*Asarum himalaicum* (Zhou et al., 2008)	Herb	0.440	NR	NR	NR	NR	NR	NR	NR	NR	NR	NR	NR	NR	NR
*Asarum sagittarioides* (Han et al., 2008; Zhou et al., 2008)	NR	0.070–0.180	NR	NR	NR	NR	NR	NR	NR	NR	NR	NR	NR	NR	NR
*Asarum sieboldii* (Wen et al., 2014; Kong et al., 2015; Zhang et al., 2016)	Root and rhizome	0.016	0.020	NR	<LOD	0.072	NR	NR	0.004–0.030	NR	NR	NR	NR	NR	NR
*Saruma henryi* (Dong et al., 2009; Zhao and Jiang, 2009; Zhang, 2011; Wang et al., 2018a)	Root	0.184–1.995	NR	NR	NR	NR	NR	NR	+	+	NR	NR	+	NR	NR
*Saruma henryi* (Zhang, 2011)	Stem	0.116	NR	NR	NR	NR	NR	NR	NR	NR	NR	NR	NR	NR	NR

The unit for quantitative data is mg/g, and the value rounds up to three decimal places.

LOD, limit of detection; LOQ, limit of quantification; N, concentration bellows 2.5 µg/ml; NR, not reported; +, present; −, absent.

**Table 2:** Contents of Aristolochic acids in Chinese patent medicine (CPM).

Name	AAs	Content	Detection method	Specific herbs in CPM
Bu fei e jiao tang (Kuo et al., 2010)	AAI, II	AAI: 119.674 AAII: 6.802	LC/MS	Herba Aristolochiae Mollissimae
Bi yan ling pian (Zhang, 2017)	AAI	−	HPLC	Radix et Rhizoma Asari
Bi yan pian (Guan et al., 2005)	AAI	3.230	HPLC	Radix et Rhizoma Asari
Chun yang zheng qi wan (Ye et al., 2003)	AAI	280	HPLC	Caulis Aristolochiae Manshuriensis
Chuan xiong cha tiao ke li (Wei et al., 2005)	AAI	+	LC/MS	Caulis Aristolochiae Manshuriensis
Chuan xiong cha tiao san (Kuo et al., 2010)	AAI	−	LC/MS	Caulis Aristolochiae Manshuriensis
Chuan xiong cha tiao wan (Ye et al., 2003)	AAI	140	HPLC	Caulis Aristolochiae Manshuriensis
Dao chi san (Wu et al., 2009)	AAI	357	HPLC	Caulis Aristolochiae Manshuriensis
Dao chi wan (Wei et al., 2005)	AAI	−	LC/MS	Caulis Akebiae
Dang gui si ni tang (Ruan et al., 2012)	AAI	321.45	HPLC	Medulla Tetrapanacis
Da huang qing wei wan (Shu et al., 2016)	AAI	0–0.08	SPE-HPLC	Caulis Akebiae
Er shi jiu wei neng xiao san (Zhang et al., 2013a)	AAI	2.69–3.71	HPLC	Fructus Aristolochiae
Er shi wu wei lv rong hao wan (Chen et al., 2009)	AAI	99–114	HPLC	Fructus Aristolochiae
Er shi wu wei shan hu wan (Liu et al., 2015)	AAI	−	HPLC, RP-HPLC	Radix Aucklandiae
Er shi wu wei shan hu wan (Luo, 2013)	AAI	52.5	HPLC	Radix Aucklandiae
Er shi wu wei song shi jiao nang (Tan et al., 2005)	AAI	0.020–0.030	HPLC	Fructus Aristolochiae
Er tong qing fei wan (Wei et al., 2005)	AAI	−	LC/MS	Radix et Rhizoma Asari
Fu fang nan xing zhi tong gao (Yin et al., 2009)	AAI	−	UPLC	Radix et Rhizoma Asari
Fu fang quan shen pian (Pang and Qu, 2015)	AAI	0.29–1.02	HPLC	Herba Aristolochiae Mollissimae
Gan lu xiao du wan (Chen and Xie, 2004; Zhu et al., 2006)	AAI, II	AAI: 60–230 AAII: 370–400	RP-HPLC	Caulis Aristolochiae Manshuriensis
Gan te ling jiao nang (Wei et al., 2007)	AAI	−	HPLC	Radix et Rhizoma Asari
Gu ben qu feng ke li (Yu et al., 2011a; Yu et al., 2011b)	AAI	−	HPLC	Radix et Rhizoma Asari
Guan xin su he di wan (Li et al., 2006; Yuan and Zhang, 2016)	AAI	148–993	HPLC	Radix Aristolochiae
Guan xin su he jiao nang (Wei et al., 2005; Li et al., 2006)	AAI, II, IIIa, IVa	AAI: 183–516 AA-II:+ AA-IIIa:+ AA-IVa:+	LC/MS	Radix Aristolochiae
Guan xin su he wan (Li et al., 2006; Wu, 2006; Jiang et al., 2007; Yuan et al., 2007a; Yuan et al., 2007b; Yuan et al., 2008)	AAI, II	AAI: 48.500–426 AAII: 64.700–65.200	RP-HPLC	Radix Aristolochiae
Han shi bi ke li (Kang and Li, 2008)	AAI	−	HPLC	Radix et Rhizoma Asari
Jian gu shu jin pian (Huang et al., 2009)	AAI	−	HPLC	Radix et Rhizoma Asari
Jiu wei qiang huo ke li (Guan et al., 2005)	AAI	1.920	RP-HPLC	Radix et Rhizoma Asari
Liu jing tou tong tablet (Huang and Zhu, 2015)	AAI	−	SPE-HPLC	Radix et Rhizoma Asari
Long dan xie gan wan (Ye et al., 2003; Liu et al., 2005; Wei et al., 2005; Shen et al., 2008)	AAI, II, IIIa, IVa,7-OH-AAI	AAI: 30–253 AAII: 44, AAIIIa: + AA-IVa: + 7-OH-AAI: +	HPLC; LC/MS	Caulis Aristolochiae Manshuriensis
	AAI, II,	−	UHPLC-MS/MS	Caulis Akebiae
Long dan xie gan ke li (Wei et al., 2005)	AAI, II, IIIa, IVa,7-OH-AAI	−	LC/MS	Caulis Akebiae
Ma huang zhi sou wan (Zhou et al., 2015)	AAI	0.070–0.210	SPE-HPLC	Radix et Rhizoma Asari
Pai shi ke li (Liu et al., 2005)	AAI, II	AAI: 4 AAII: 4	SPE-HPLC HPLC	Caulis Akebiae
Qing nao zhi tong jiao nang (Li et al., 2009)	AAI	−	HPLC	Radix et Rhizoma Asari
Qing ning wan (Ye et al., 2003)	AAI	100	HPLC	Caulis Aristolochiae Manshuriensis
Qing lin ke li (Yuan et al., 2007a; Yuan et al., 2007b; Yuan et al., 2008)	AAI, II, IVa;AL-IV,AL-IVa	AAI: 114–184 AAII: 56.200–62.400 AAIVa: 40.800–58.200 ALIV: 52.500 ALIVa: 32.500–160	HPLC LC/MS	Caulis Akebiae
Qi wei hong hua shu sheng wan (Wei et al., 2011)	AAI	241–385	HPLC	Fructus Aristolochiae
Qing xue nei xiao wan (Gan et al., 2013)	AAI	+	HPLC LC/MS	Caulis Akebiae
Ru mo zhen tong jiao nang (Xue, 2015)	AAI	−	HPLC	Radix et Rhizoma Asari
Shen nong she yao jiu (Qiu et al., 2018)	AAI	+	HPLC	*Aristolochia fordiana*
Tiao gu pian (Lin et al., 2014)	AAI	2.860–6.250	HPLC	Radix et Rhizoma Asari
Wan tong jin gu pian (Tian and Wang, 2007)	AAI	2.403–4.779	RP-HPLC	Radix et Rhizoma Asari
Wu wei zha xun wan (Ni and Yang, 2012)	AAI	260–280	HPLC	Fructus Aristolochiae
Xiao feng zhi yang ke li (Zhang and Wang, 2012)	AAI	−	LC-MS	Caulis Akebiae
Xiao qing long ke li (Guan et al., 2005)	AAI	2.86	RP-HPLC	Radix et Rhizoma Asari
Xiao qing long tang (Kuo et al., 2010)	AAI	0.194	LC/MS	Radix et Rhizoma Asari
Xiao Zhong zhi tong ding (Tang et al., 2012; He et al., 2013)	AAI	+	LC-MS/MS, SPE-HPLC	Radix et Rhizoma Asari
Xiao Zhong zhi tong ting (Tang et al., 2012; He et al., 2013)	AAI	+	HPLC	Radix et Rhizoma Asari
Xin ma zhi ke ke li (Chen et al., 2007)	AAI	−	HPLC	Radix et Rhizoma Asari
Xin qin ke li (Ge et al., 2010)	AAI	−	HPLC, SPE-HPLC	Radix et Rhizoma Asari
Xin sheng ke li (Ren et al., 2010)	AAI	−	HPLC	Radix et Rhizoma Asari
Xi xin pei fang ke li (Pan and Gan, 2009)	AAI	−	LC-MS/MS	Radix et Rhizoma Asari
Yang xue qing nao ke li (Li et al., 2007)	AAI	−	HPLC, LC-MS	Radix et Rhizoma Asari
Yang yin jiang ya jiao nang (Ran et al., 2016)	AAI	1129–1458	HPLC	Radix Aristolochiae
Yi shen juan bi wan (Wang et al., 2017)	AAI	−	HPLC	Herba Aristolochiae Mollissimae
Zhui feng tou gu capsule (Lv, 2015)	AAI	−	HPLC	Radix et Rhizoma Asari
Zhu sha lian jiao nang (Chen, 2003)	AAI	1160–4521	HPLC	Radix Aristolochiae Cinnabarinae

The unit for quantitative data is µg/g.

## Aristolochic Acid-Induced Adverse Reactions

### Aristolochic Acid Nephropathy

AA nephropathy (AAN) is a kind of chronic tubulointerstitial renal disease accompanied by upper tract urothelial carcinoma (UTUC) in almost half of the cases (Nortier et al., 2000). In 1992, some female patients from Belgium who consumed slimming pills containing Chinese herbs suffered from rapidly progressive interstitial nephritis (Vanherweghem et al., 1993). The renal failure was characterized by extensive interstitial fibrosis with atrophy, loss of tubules, and hyperplasia of the urothelium mainly localized in the superficial cortex (Cosyns et al., 1994a; Depierreux et al., 1994). Thereafter, urothelial carcinoma occurred in more than 40% of the patients consuming these Chinese herbs (Cosyns et al., 1994b; Nortier et al., 2000; Lord et al., 2001; Nortier and Vanherweghem, 2002). After investigations, it was found that *Stephania tetranda* was inadvertently substituted by *Aristolochia fangchi*, which contained nephrotoxic constituents (AAs) leading to adverse events (Vanhaelen et al., 1994). AAs were substantiated as the chief culprit because AA-derived DNA adducts were detected in the kidneys and ureteric tissues of these patients (Schmeiser et al., 1996; Nortier et al., 2000; Lord et al., 2001). Since then, AAN has raised worldwide attention (Jadot et al., 2017). Long-term ingestion of herbal formula known or suspected to contain AAs is one of the prominent risk factors for developing AAN (Jia et al., 2005; Vervaet et al., 2017). Although the sale and use of AA-containing products are banned or restricted in most of the countries (Krell and Stebbing, 2013), AAN induced by numerous herbal remedies and products are still reported from all over the world (Lord et al., 1999; Yang et al., 2006; Debelle et al., 2008; Shaohua et al., 2010; Wu et al., 2012; Vaclavik et al., 2014; Ban et al., 2018).

Generally, most AAN patients display an unusually rapid progression towards end-stage renal disease. During clinical examination, mild hypertension, severe anemia, increased serum creatinine, decreased estimated glomerular filtration rate, proteinuria, glycosuria, and/or leukocyturia may be observed in most cases (Reginster et al., 1997; Meyer et al., 2000; Yang et al., 2012; Gokmen et al., 2013). More precisely, some studies reveal that microalbuminuria and proteinuria of tubular type can serve as early screening indicators of AAN (Kabanda et al., 1995; Trnacevic et al., 2017). Estimation of neutral endopeptidase, a 94-kDa ectoenzyme of the proximal tubule brush border, which is characteristically decreased in AAN patients, may also serve as an early clinical biomarker of AAN (Nortier et al., 1997). During renal tract ultrasonic inspection, shrunken kidneys are observed, which results in asymmetrical and irregular cortical outline (Gokmen et al., 2013). Microscopically, the typical findings are extensive interstitial fibrosis with atrophy and loss of tubules localized predominantly in the superficial cortex and progressing towards the inner cortex. The interstitium is remarkably hypocellular in a majority of the cases. Interstitial inflammatory infiltration is observed in some cases, and more inflammatory cells are found as compared with other renal diseases. The glomeruli are relatively spared. The collapse of the capillaries and wrinkling of the basement membrane are noticed in a few glomeruli. Glomerular lesions mainly include ischemic, microcystic, obsolescent glomeruli, occasional thrombotic microangiopathy-like lesions, and/or focal segmental sclerosis-like lesions. Multifocal thickening of interlobular and afferent arterioles and/or splitting up of peritubular capillary basement membranes may be observed, which are associated with arteriolar hyalinosis, intimal fibrous hyperplasia, and occasional mucoid arterial intimal fibrosis (Depierreux et al., 1994; Meyer et al., 2000; Stefanovic et al., 2007; Debelle et al., 2008; Jelakovic et al., 2014; Jadot et al., 2017).

### Balkan Endemic Nephropathy

After AAs were substantiated as one of the main causative agents inducing rapidly progressive renal disease, some scientists proposed that the clinical and morphological features of different stages of AAN and the patterns of the famous BEN were strikingly similar. This provided a clue that BEN may also be related to AAs (Cosyns et al., 1994a; Arlt et al., 2002; Grollman et al., 2007; de Jonge and Vanrenterghem, 2008). BEN is an endemic familial but not inherited chronic renal disease, which is frequently accompanied by urothelial carcinoma of the upper urinary tract (Stefanovic et al., 2007; Miyazaki and Nishiyama, 2017). The disease is prevalent in the endemic farming villages along the tributaries of the Danube river (Stiborova et al., 2016). It has been estimated that almost 25,000 people have caught this disease, and nearly 100,000 people are still at risk (Bamias and Boletis, 2008; Pavlovic, 2013a).

Several hypotheses on the etiology of BEN have been projected in the past decades, including mycotoxins, phytotoxins, heavy metals, viruses, trace element deficiencies, and AAs (Stefanovic et al., 2006; Grollman et al., 2007; De Broe, 2012). Among these, the evidence is strongest for inadvertent chronic consumption of food contaminated with AAs leading to BEN (Grollman et al., 2007; De Broe, 2012; Bui-Klimke and Wu, 2014). Researchers assume that the BEN patients might be exposed to toxic AAs through consuming food prepared from flour contaminated with the seeds of the plants of *Aristolochia* family, which grow abundantly as weeds in the endemic regions (Ivic, 1969; Hranjec et al., 2005; Jelakovic et al., 2012; Grollman, 2013; Jelakovic et al., 2015b). Moreover, molecular agricultural and food chemistry investigations have been carried out to trace other possibilities on how AAs enter the human food chain. Studies have demonstrated that some crops could uptake and bioaccumulate AAs from the *Aristolochia* species-grown soil and water. Therefore, prolonged intake of food prepared from these crops can also result in BEN (Pavlovic et al., 2013b; Chan et al., 2016; Li et al., 2016; Gruia et al., 2018). In recent years, the definitive link between BEN and AAs has been found. AA-derived DNA adducts and hallmark A→T transversions have been detected in renal cortical and urothelial malignant tissues obtained from BEN patients (Grollman et al., 2007; Jelakovic et al., 2012).

### Aristolochic Acid-Induced Urothelial Carcinoma

Apart from nephrotoxicity, AAs have also been implicated in the genesis of UTUC, which is a rare subset of urothelial malignancies occurring in the renal pelvis and upper ureter. UTUC has been so far principally correlated to AA intoxication (Miyazaki and Nishiyama, 2017). At first, scientists found progressive urothelial atypia and atypical hyperplasia in the tissue samples of Belgian patients diagnosed with slimming pill-induced nephrotoxicity (Cosyns et al., 1994a; Cosyns et al., 1994b). Subsequently, urothelial carcinoma localized in the upper urinary tract was observed in almost half of the AAN patients (Nortier et al., 2000). AA-derived DNA adducts and *TP53* mutations were also found in the ureteric tissues (Cosyns et al., 1999; Lord et al., 2001), which indicated the carcinogenic potential of AAs on the urothelium. In addition, the prevalence of UTUC is extraordinarily high in BEN patients (Colin et al., 2009; Patel et al., 2014; Soria et al., 2017). The data on AA-derived DNA adducts and A→T transversions further corroborated the relationship between AAs and BEN-associated urothelial tumor (Arlt et al., 2002; Arlt et al., 2007; Schmeiser et al., 2012).

Moreover, the trend of urothelial cancer among the patients diagnosed with end-stage renal disease has been reduced with the decreased consumption of AA-containing products (Wang et al., 2014a; Wang et al., 2014b). In 2002 and 2012, the World Health Organization International Agency for Research on Cancer (IARC) classified AAs as group I carcinogen according to the available strong evidence that AA-specific DNA adducts and *TP53* mutations were found in humans exposed to materials obtained from plant species containing AAs (IARC, 2002; IARC, 2012). However, despite a high mutagenic and carcinogenic potential, herbal remedies and products containing AAs are still used in Asia contributing to a high incidence of urothelial carcinoma (Chen et al., 2012; Yang et al., 2014; Sun et al., 2015).

Most cases of AA-induced UTUC are found during AAN inspections. Initially, only mild to moderate urothelial atypia and atypical hyperplasia were observed (Cosyns et al., 1994a). Subsequently, overwhelming disseminated pelvicalyceal urothelial atypia, malignant transformation, and/or multifocal flat transitional cell carcinoma mostly localized in the upper urinary tract were shown (Cosyns et al., 1994b; Cosyns et al., 1999). Usually, these tumors have a high mortality rate. The urothelial carcinomas are mainly of synchronous bilateral or metachronous contralateral type and are related to the cumulative exposure of AAs (Chen et al., 2013; Sun et al., 2015). In addition, AA-derived DNA adducts and *TP53* mutations are clinically meaningful to explore the involvement of AAs in UTUC (Chen et al., 2012; Chen et al., 2013; Aydin et al., 2014; Yang et al., 2014; Sun et al., 2015).

### Other Adverse Effects Induced by Aristolochic Acids

Besides UTUC, AA-mutational signatures have also been detected in other types of cancer, which indicates that AAs also display carcinogenic potentials in other organs (Rosenquist and Grollman, 2016). Clinically, patients with hepatitis B virus infection are presumed to have a higher risk of hepatocellular carcinoma if they consume AA-containing herbs (Chen et al., 2018). Genomic heterogeneity analyses provide strong evidence that AAs potentially contribute to the development of liver cancer (Poon et al., 2013; Lin et al., 2017). Recently, a specific mutational signature of AA exposure has been exhibited in whole exome sequencing of hepatocellular carcinomas, suggesting a plausible conclusion that AAs and their derivatives might be one of the culprits triggering liver cancer in Asia (Totoki et al., 2014; Letouze et al., 2017; Ng et al., 2017; Nault and Letouze, 2019). AAs can also affect the initiation and/or progression of renal cell carcinoma (Scelo et al., 2014; Jelakovic et al., 2015a; Hoang et al., 2016) or bladder urothelial tumor (Lemy et al., 2008; Poon et al., 2015; Sun et al., 2015).

More carcinogenic potentials and/or toxic effects of AAs are explored in animal studies. High risk of tumor occurrence in the fore-stomach, ear duct, small intestine, kidney, urothelial tract, liver, bladder, and/or subcutaneous regions were observed in mice, rats, and/or canines after AA administration (Mengs, 1982; Mengs, 1988; Schmeiser et al., 1990; Wang et al., 2011; Wang et al., 2018b; Jin et al., 2016). Renal toxicity of AAs is observed in both mice and rats after repeat dose (Mengs, 1987; Mengs and Stotzem, 1993). Furthermore, aristolochic acid I (AAI)-induced gastrotoxicity characterized by fore-stomach damage presents prior to renal injury (Pu et al., 2016). In addition, AAI could induce apoptotic cell death in the ovaries and testis of mice and cause severe reduction of organ size and weight (Kwak et al., 2014; Kwak and Lee, 2016).

## Toxicological Properties of Aristolochic Acids

### Mutational Signature of Aristolochic Acids

As mentioned above, AAs are conversed to reactive intermediates (aristolactam nitrenium ion) and bind to purines in DNA to form covalent DNA adducts. The adducts of AAs with DNA are highly persistent in human tissues (Schmeiser et al., 2014) due to the lack of recognition and/or processing by global genome nucleotide excision repair (Lukin et al., 2012; Sidorenko et al., 2012). Without effective DNA repair, predominant A→T transversions enriched on the nontranscribed gene strand in the *TP53* tumor suppressor gene could form in high frequency (Moriya et al., 2011; Hoang et al., 2013). The *TP53* mutations and formation of AA-derived DNA adducts are considered as biomarkers for the assessment of AA exposure (Slade et al., 2009; Stiborova et al., 2017). Mutated base adenine accounts for more than half of the mutational spectra detected in the specimens of AAN and AA-induced UTUC patients (Lord et al., 2004; Chen et al., 2012; Jelakovic et al., 2012; Hoang et al., 2013; Castells et al., 2015). Besides A→T transversions, C→T transversions also occur in a high frequency. Multiple mutations are mainly found in the *TP53* hotspot region of exons 5–8, as well as exons 4 and 10 (Moriya et al., 2011; Aydin et al., 2017). In animal studies, A→T transversions were observed in the activating positions in H-*ras* of rats treated with AAs (Wang et al., 2011). Further, RNAs modified by AAs were at much higher frequencies than DNA (Leung and Chan, 2015).

### Biotransformation of Aristolochic Acids

**Figure 2:**
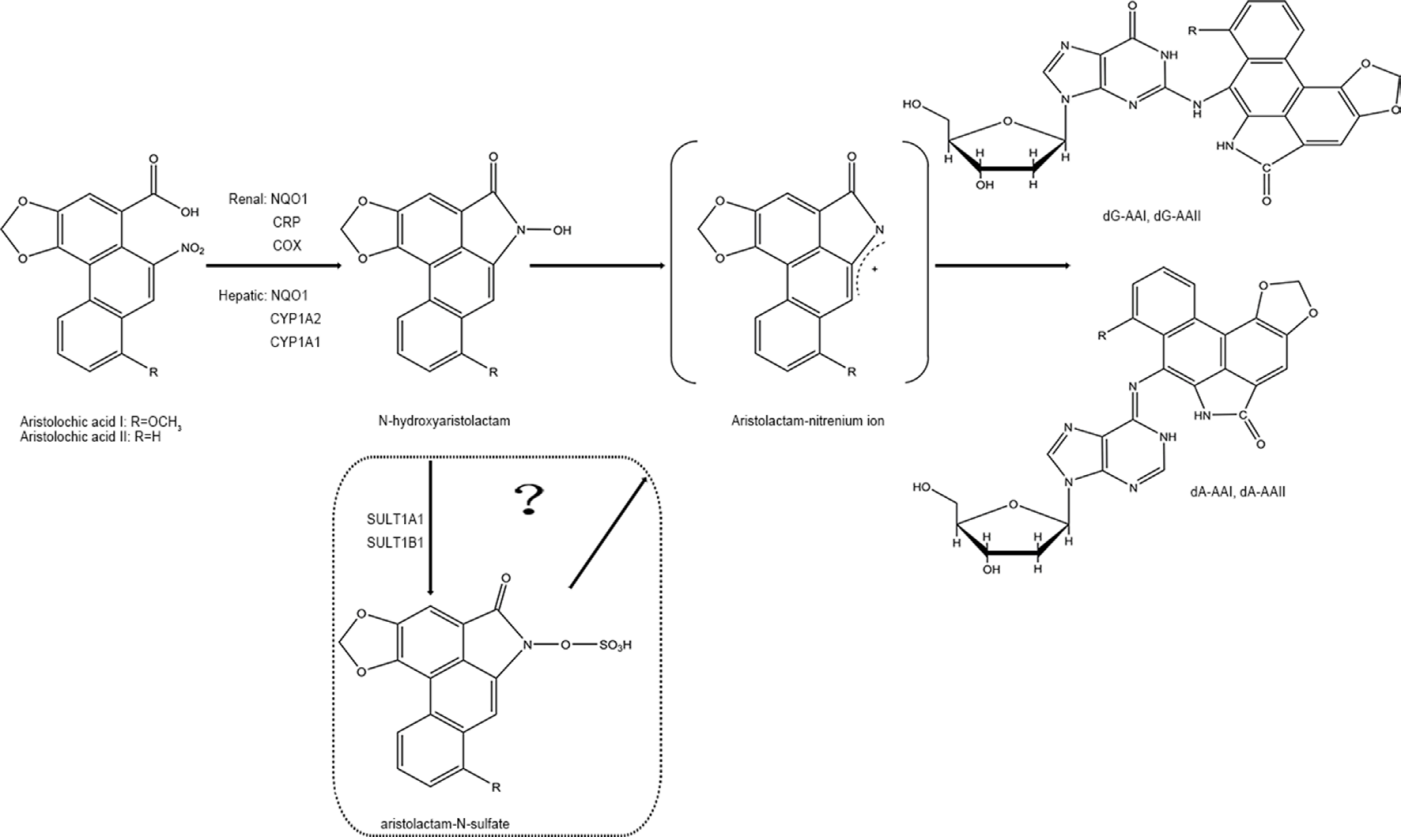
Proposed pathway for metabolic activation of AAI and AAII.

In phase I biotransformation reaction, AAs are first transformed to N-hydroxyaristolactams (AL-NOHs) through nitroreduction reaction. After that, they are converted to aristolactam-nitrenium, which is an electrophilic cyclic aristolactam-nitrenium ion with delocalized positive charges. They preferentially bind to the exocyclic amino groups of purine bases in DNA to form AA-DNA adducts. These adducts can lead to A→T transversions and elicit renal disease and cancers (Stiborova et al., 2008b; Stiborova et al., 2008c). Both microsomal and cytosolic phase I enzymes participate in catalyzing the activation of AAs to form AA-DNA adducts consisting of 7-(deoxyadenosin-N^6^-yl) aristolactam I (dA-AAI), 7-(deoxyguanosin-N^2^-yl) aristolactam I (dG-AAI), 7-(deoxyadenosin-N^6^-yl) aristolactam II (dA-AAII), and 7-(deoxyguanosin-N^2^-yl) aristolactam II (dG-AAII) (Stiborova et al., 2005; Stiborova et al., 2008a), in which dA-AAI and dA-AAII persist for an exceptionally long time in the lesions (Lukin et al., 2012; Schmeiser et al., 2014). The dA adducts are also significantly more mutagenic than the dG adducts (Attaluri et al., 2010). According to the results from *in vitro* studies, among the cytosolic reductases, NAD(P)H:quinone oxidoreductase (NQO1) plays the most important role in activation of AAs in the liver and kidney. In human renal microsomes, NADPH : CYP reductase (CPR) is proven to be more effective in activating AAs, and prostaglandin H synthase (cyclooxygenase, COX) is also involved in the reductive reaction. In human hepatic microsomes, CYP1A2 contributes maximum in the process, while CYP1A1 exhibits lesser effects and CPR plays a minor role (Stiborova et al., 2005; Stiborova et al., 2008a). Currently, the roles of the hydroxyl group on amino acids present in the active center of CYP1A1 and CYP1A2 on nitroreduction of AAs have been verified using a site-directed mutagenesis approach (Milichovsky et al., 2016). It is assumed that the genes of enzymes existing in variant forms or showing polymorphisms may be one of the factors affecting the individual’s susceptibility to AAs (Stiborova et al., 2008a). Additionally, phase II metabolisms formed by sulfation are reported to readily produce AA-DNA adducts (Sidorenko et al., 2014). It has been predicted that following reductive reactions, AL-NOHs may serve as substrates for sulfotransferases (SULTs) and convert them to N-sulfated esters, which react more efficiently with DNA (Sidorenko et al., 2014; Hashimoto et al., 2016). Among the SULTs tested, SULT1A1 and SULT1B1 displayed more activity than the other subtypes (Meinl et al., 2006; Sidorenko et al., 2014). The sulfate conjugates could be transported out of the liver *via* MRP membrane transporters and transferred into the kidney *via* organic anionic transporters (OAT), thereby inducing kidney damage (Chang et al., 2017). However, conﬂicting results have been observed in some other studies (Stiborova et al., 2011; Arlt et al., 2017) (**Figure 2**).

**Figure 3:**
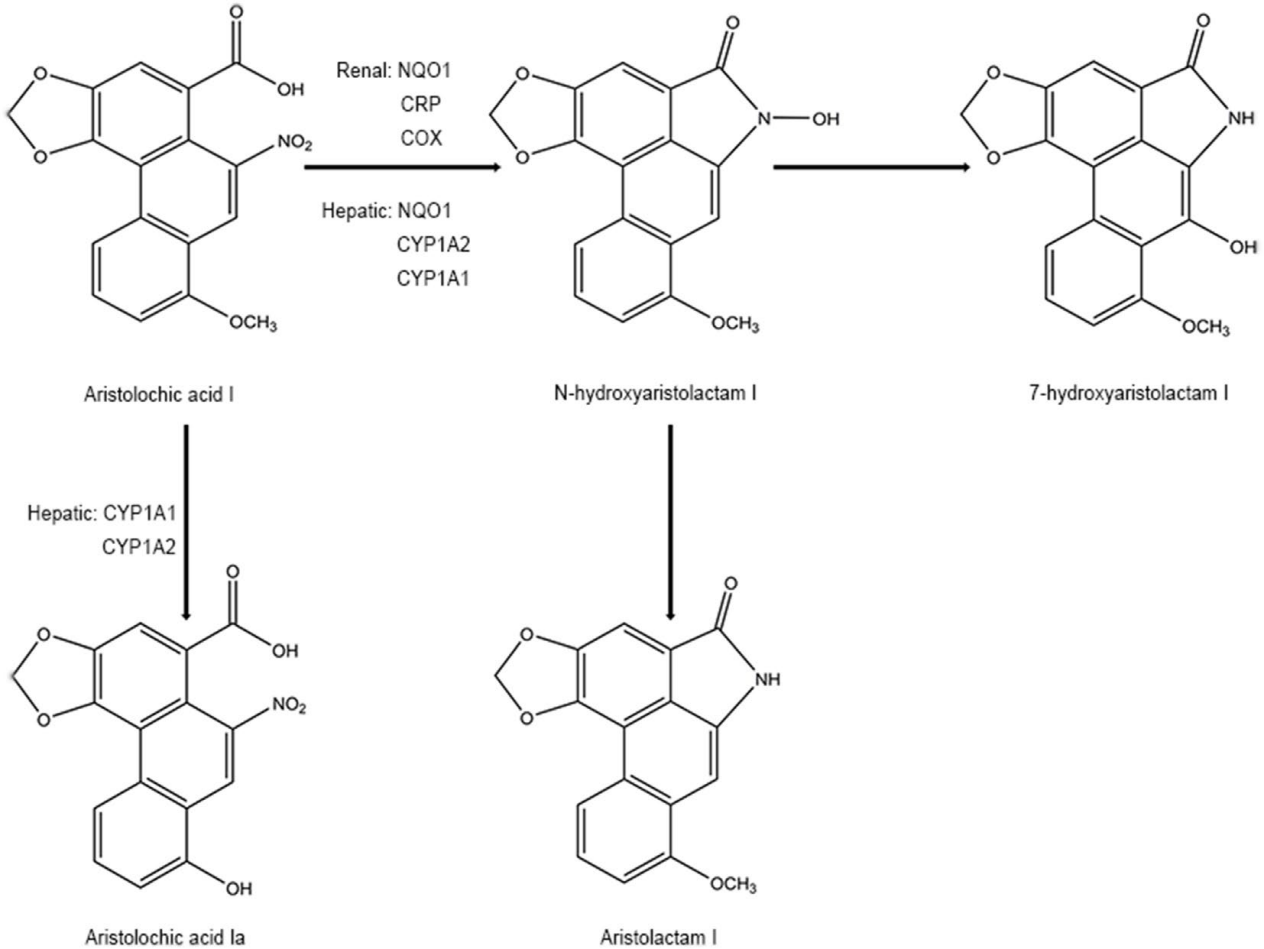
Proposed pathway for metabolic detoxication of AAI.

On the other hand, detoxification of AAI happens at the same time when it exerts mutagenic and cytotoxic potentials. Some studies have mentioned that N-hydroxyaristolactam I could also competitively rearrange to 7-hydroxyaristolactam I or further reductive production of aristolactam I (ALI) (Stiborova et al., 2008b; Stiborova et al., 2008c), which shows a lower capacity to form AAI-DNA adducts (Dong et al., 2006). In addition, oxidation of AAI to a lesser toxic 8-hydroxyaristolochic acid I (aristolochic acid Ia, AAIa) is also suggested to be a detoxifying pathway of AAI (Stiborova et al., 2008b; Stiborova et al., 2008c). The O-demethylated metabolites of AAI, conjugated metabolites of AAIa, are found to be excreted in the urine of AA-treated rats (Chan et al., 2007). Hepatic microsomal cytochrome P450, especially CYP1A subfamily (CYP1A1 and CYP1A2), has been found to play a critical role in suppressing the carcinogenic and nephrotoxic effects of AAI (Xiao et al., 2008; Stiborova et al., 2009; Rosenquist et al., 2010; Stiborova et al., 2012; Stiborova et al., 2015; Dracinska et al., 2016). Therefore, the CYP1A1 and 1A2 play a dual role by partly regulating the balance between reductive activation and oxidative detoxification of AAI (**Figure 3**). However, the analogical mechanism has not been observed in AAII yet because AAII shows much lower amenability to oxidation than AAI (Martinek et al., 2017).

### Specific Organic Anion Transporters for Aristolochic Acids

The nephrotoxic damage of AAs selectively targets the proximal tubules, indicating that the toxins may specifically accumulate in these tissues. The proximal tubules take charge in the secretion and reabsorption of xenobiotics or their metabolites through several particular transporters. OAT family, a group of multispecific membrane transport proteins, contributes to the renal handling of negatively charged drugs and other organic compounds. Indeed, AAI as a low molecular weight organic anion with an anionic carboxyl group and a hydrophobic part possesses the chemical characteristics of a substrate for OAT. OAT family is, therefore, considered to be one of the pivotal determinants mediating the accumulation of AAI into the renal proximal tubules (Dickman et al., 2011). Many investigations have verified OATs, especially OAT1 and OAT3, in the basolateral membrane of the proximal tubules facilitating the uptake of AAI by renal cells, which at least partly lead to site-selective AAI-induced nephrotoxicity (Bakhiya et al., 2009; Dickman et al., 2011; Xue et al., 2011; Baudoux et al., 2012). In addition, the phase II metabolite of AAI, sulfate-conjugated AL-I (sulfonyloxyaristolactam, AL-I-NOSO_3_) is reported to be transported into kidney *via* OAT1, OAT3, and OAT4 (Chang et al., 2017).

### Other Mechanisms Involved in Aristolochic Acid-Induced Adverse Reactions

The chemical structures of AAs are considered as critical determinants of their toxic effects. According to the current knowledge, AAI is solely responsible for nephrotoxicity (Shibutani et al., 2007) and has more cytotoxicity than AAII (Balachandran et al., 2005). On the other hand, AAII may show higher or similar genotoxic and carcinogenic potentials as AAI (Shibutani et al., 2007; Xing et al., 2012). In reductive reactions, NQO1 is more effective in activating of AAI than AAII (Martinek et al., 2011), and the extent of AAI-DNA adducts is much higher than that of AAII-DNA adducts in most *in vitro* enzymatic systems (Schmeiser et al., 1997). During phase II metabolism, ALI-DNA adducts are also formed more efficiently than ALII-DNA adducts in SULT1B1 (Sidorenko et al., 2014). In addition, similar CYP-mediated oxidative detoxification reactions of AAI are not observed in AAII (Martinek et al., 2017). The difference on enzymatic conversion of AAI and AAII is considered to relate to their chemical structures.

In recent years, more than 30 microRNAs differentially expressed in patients exposed to AAs have been explored, which might improve the understanding of the pathogenesis of AA-related renal disease and cancer. It has been speculated that FGFR3, Akt, mucin type O-glycan biosynthesis, ECM receptor interaction pathways, and other biological mechanisms might be involved in the occurrences of AAN, BEN, and/or AA-induced UTUC (Tao et al., 2015; Lv et al., 2016; Popovska-Jankovic et al., 2016). Meanwhile, 15% of the 417 detectable miRNAs have been found to be altered by AAs in rats (Li et al., 2015). During exome sequencing of genes in DNA samples from BEN patients, possible deleterious/damaging variants—*CELA1*, *HSPG2*, and *KCNK5*—are detected. These genes could encode proteins connected to the process of angiogenesis (Toncheva et al., 2014), which is tightly correlated with BEN and UTUC (Jankovic-Velickovic et al., 2012).

Some studies have focused on the contributions of innate and adaptive immunity in the progression of AAN (Sato et al., 2004; Pozdzik et al., 2010). In the kidney specimens, interstitial inflammatory infiltration is dramatically observed in the tubulointerstitial lesions, and massive infiltration of macrophages and T and B lymphocytes is demonstrated in the medullary rays and outer medullae, implying the onset of immune response (Pozdzik et al., 2010). Animal study has suggested that AAs increase the proportion of myeloid CD11b^high^F4/80^mid^ and decrease their counterpart. CD4^+^ and CD8^+^ T-cells could provide protection against AA-induced acute tubular necrosis (Baudoux et al., 2018). However, this view is still debatable as AAI is also reported to damage the epithelial cells lining the proximal renal tubule due to direct toxic eﬀects instead of immune actions (Yi et al., 2018).

Meanwhile, AAs could cause increased oxidative stress leading to impaired renal function. AAI elicits oxidative stress-related DNA damage through depleting antioxidant glutathione in the human renal proximal tubular cells (Yu et al., 2011a; Yu et al., 2011b) and induces increased reactive oxygen species (ROS) and tubular apoptosis *via* decreasing nitric oxide availability in mice (Decleves et al., 2016).

Inﬂammatory and fibrotic pathways and dynamic changes in fatty acid, phospholipid, and glycerolipid metabolisms are all linked to AAN (Zhao et al., 2015). Additionally, mitogen-activated protein kinase (MAPK)-related signaling pathways are considered to be associated with nephrotoxicity and reproductive toxicity of AAI. AAI could upregulate the expression of phospho-ERK1/2 in cells, which contributes to ROS generation (Yu et al., 2011a; Yu et al., 2011b). AAs also activate JNK signaling pathway and elicit the overexpression of TGF-1, which is critically involved in the pathogenesis of AAN (Rui et al., 2012). Interestingly, some other studies have described that AAI inhibits Akt and/or ERK1/2 phosphorylation, impedes relevant apoptosis, and causes severe injury resulting in the development of ovarian and testis in mice (Kwak et al., 2014; Kwak and Lee, 2016; Yu et al., 2011b). Besides, NFκb, aryl hydrocarbon receptor, and cell cycle signaling are also modulated in the kidneys of AA-treated mice (Arlt et al., 2011). In addition, the loss of functional TASK-2 channels may indirectly increase the susceptibility to the toxic effects of AAs (Veale and Mathie, 2016). The decrease in pro-apoptotic protein Bax can predict the development of AA-induced UTUC (Jankovic-Velickovic et al., 2011).

## Conclusion

AAs have been recognized as a group of potent nephrotoxin and carcinogen. The consumption of AA-containing foods can cause permanent kidney injury, end-stage renal disease, and even UTUC. AA-derived DNA adducts are recognized as specific biomarkers for the assessment of AA exposure. A characteristically mutational signature of A→T transversions observed in the tumor tissues also implies the exposure of AAs. So far, the underlying etiological mechanisms of AA-induced renal disease and UTUC have been preliminarily revealed, although the detailed mechanisms are far from being completely understood. In addition, various enzymes, organic anion transporters, and molecular mechanisms might be involved in AA-induced damages. Therapy for AAN and AA-induced UTUC remains a serious challenge. AA-related adverse events are still widely reported, especially in the Asian and Balkan regions. AAs have now been listed as group I carcinogen by IARC (IARC, 2002; IARC, 2012). The US Food and Drug Administration and regulatory authorities of some other countries have issued alerts against the use and import of products containing AAs (Zhang et al., 2019). However, in China and some countries, products containing herb preparations from *Aristolochia* and *Asarum* are still used, and food contaminated by AAs cause health problems in some countries (Ioset et al., 2003; Wooltorton, 2004; Hsieh et al., 2008; Michl et al., 2013; Ardalan et al., 2015; Cachet et al., 2016; Abdullah et al., 2017). Therefore, the use of AA-containing herbal medications and consumption of food contaminated by AAs may still impose high risk, and hence, more strict precautions should be taken to protect the public from AA exposure.

## Author Contributions

JH, ZX, and JL are the major writers of the manuscript. YZ has drawn the pictures. AL has overseen the writing.

## Funding

This work was supported by grants from the National Science and Technology Major Project (2015ZX09501004-003-001 and 2018ZX09101002-003), Beijing Science and Technology Projects (Z151100000115012 and Z161100004916025), and China Academy of Chinese Medical Sciences Foundation (ZZ10-025 and ZZ12-001).

## Conflict of Interest Statement

The authors declare that the research was conducted in the absence of any commercial or financial relationships that could be construed as a potential conflict of interest.
